# Retrosternal benign ectopic thyroid tissue: A case report with literature review

**DOI:** 10.1016/j.radcr.2025.06.051

**Published:** 2025-07-17

**Authors:** Mohsen Bashammakh, Turki Almuhaimid, Abdulhadi Almutairi, Reem AlHubail

**Affiliations:** a Faculty of Medicine, Imam Abdulrahman Bin Faisal University, Dammam, & King Fahad Hospital of the University, Al-Khobar, Saudi Arabia; b King Fahad Specialist Hospital, Dammam, Saudi Arabia

**Keywords:** Ectopic thyroid tissue, Benign thyroid mass, Superior mediastinal mass, Retrosternal mass, case report

## Abstract

Ectopic thyroid tissue is an uncommon migratory anomaly of the thyroid, occurring in 1 in 100,000 to 300,000 individuals, with a greater prevalence in females. Although lingual ectopic thyroid represents 90% of cases, extralingual sites, such as the mediastinum, are less common. Despite being frequently asymptomatic, it can present with dysphagia and dyspnea because of mass impact. A 27-year-old Saudi woman with progressive dysphagia and intermittent. Imaging demonstrates a well-defined retrosternal mass distinct from the native thyroid gland. Fine-needle aspiration verified benign multinodular thyroid tissue. A successful surgical excision was conducted by a low cervical approach without the need for sternotomy. Histopathology confirmed benign ectopic thyroid tissue, and the patient had an uncomplicated recovery with normal thyroid function postoperatively. This case highlights the significance of considering Ectopic thyroid tissue in the differential diagnosis of anterior neck masses causing dysphagia and contributes to the limited literature on ectopic thyroid presentations.

## Background

In normal development, the thyroid gland travels from the base of the tongue to the pretracheal location [1]. Ectopic thyroid tissue (ETT) is a rare thyroid migratory aberration that affects 1 in 100,000 to 300,000 with a higher prevalence in females. [2,3]. While lingual ectopic thyroid accounts for 90% of incidences, extralingual locations like the mediastinum, axilla, esophagus, gallbladder, and duodenum are rarer [2,3]. Although usually asymptomatic, mass effect symptoms like dysphagia and dyspnea may occur [2,3]. We present a case of benign ETT presenting as an anterior neck mass causing dysphagia in a young woman.

## Case report

A 27-year-old Saudi woman otherwise healthy presented in August 2024 with a history of progressive solid food dysphagia and intermittent dyspnea for 6 months. She had received treatment for acid reflux without improvement and reported occasional headaches and dizziness without identifiable triggers. Examination revealed stable vital signs, a normal anterior neck, and no palpable mass or signs of systemic illness.

**Fig. 1:**
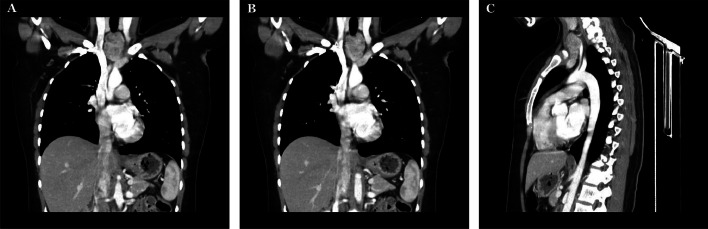
A coronal chest CT showing a retrosternal mass that is separated from the thyroid gland by fatty tissue and is attached to the great vessels (A and B). A sagittal chest CT reveals the mass’s retrosternal extensions and attachment to the superior vena cava (SVC) (C).

**Fig. 2:**
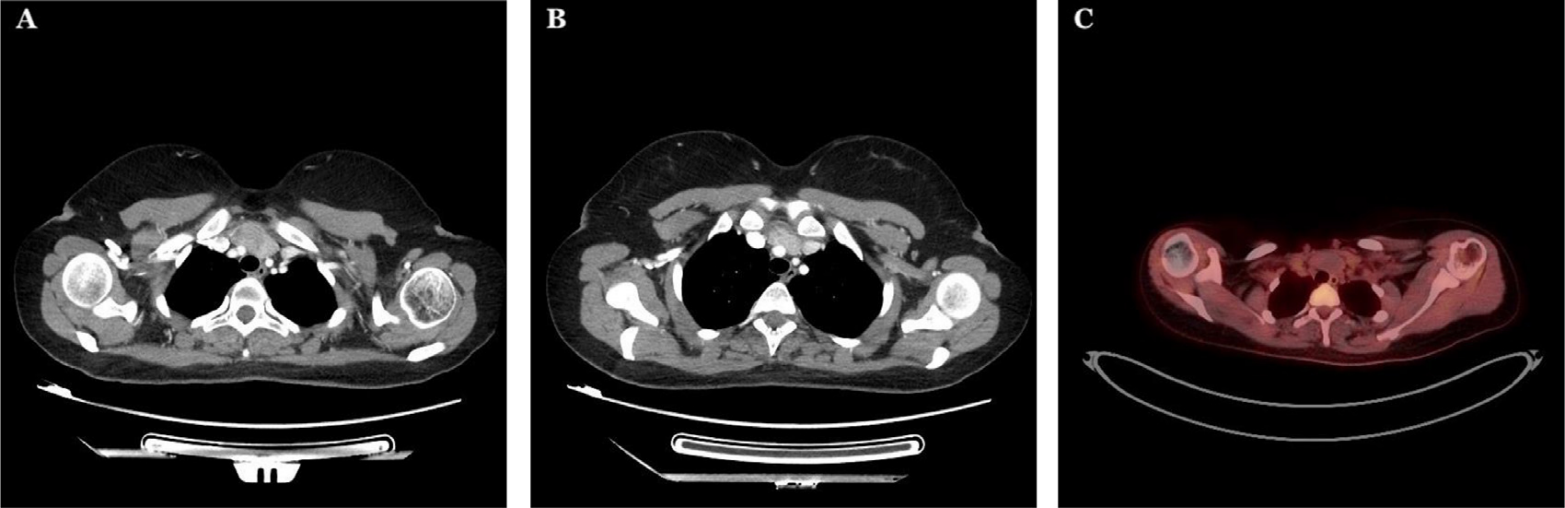
An axial CT showing part of the mass that is attached to the superior vena cava and innominate artery (A and B). An axial PET scan mass showing no observed uptake or metabolic activity (C).

Thyroid function tests (TFTs), including T4 and TSH were within normal limits. Upper Gastrointestinal endoscopy and flexible laryngopharyngoscopy excluded gastrointestinal causes. A contrasted computed tomography (CT) scan of the head and neck revealed a well-localized retrosternal soft tissue mass distinct from the main thyroid gland without infiltrating surrounding structures (Fig. 1, Fig. 2). A positron emission tomography (PET) scan ruled out thymoma and myasthenia gravis (Fig. 2). Ultrasound-guided fine-needle aspiration (FNA) of the mass showed benign multinodular thyroid tissue of ectopic thyroid origin. Gross examination found 3 reddish-yellow, thin, fragmented cores measuring between 0.2 cm and 0.7 cm, and microscopic analysis verified the presence of benign thyroid tissue. Also, positive thyroid transcription factor-1 (TTF-1) staining confirmed thyroid tissue origin, whereas negative markers for chromogranin and GATA Binding Protein 3 (GATA-3) excluded neuroendocrine and nonthyroid tissue involvement.

Because of the mass’s excellent placement, a low cervical incision was performed. While the patient was supine with neck extension on the operating table, the superior portion of the tumor was palpated before the operation, despite being nonpalpable on clinical neck examination. The surgery was completed without complications, and the entire ectopic thyroid mass was excised without sternotomy or manubriotomy. Pathological examination confirmed a single piece of solid and cystic mass measuring 4.5 × 3.0 × 1.0 cm. The final diagnosis was benign thyroid tissue with no evidence of malignancy. The patient’s postoperative course was uneventful. She was discharged on October 1, 2024, and her pain was managed with paracetamol and tramadol. On the first follow-up visit, the patient reported significant improvement without complications noted. TFTs remained normal postoperation. This evaluation was essential to rule out hypothyroidism, as there was a concern that the excised ETT might have been the patient’s only functioning thyroid tissue. TFTs were repeated to monitor for any postoperative alterations.

## Discussion

In the literature, the age and gender distribution differed among different studies. Pilavaki et al. described a 72-year-old male, whereas Hummel et al. reported a 61-year-old female [1,4]. Nagireddy et al. documented a case of a 57-year-old woman, and Melinte et al. involved a 40-year-old man [5,6]. Muzurović, Regal, El Haj, Imai, and Mace documented cases in females aged 53, 32, 59, 50, and 80 years, respectively [2,[7], [8], [9], [10]]. Wu et al. and Truong et al. described females aged 55 and 50, while Kola et al. and Walz et al. presented males aged 42 and 31 [3,[11], [12], [13]]. Our patient, being younger than most cases documented, perhaps attributable to early detection. The gender distribution indicates a female majority, suggesting possible predisposition factors.

Our patient's dysphagia and dyspnea are consistent with prior cases in the literature. Hummel et al. reported cough and chest pain, whereas Nagireddy et al. saw chest discomfort and cough [1,5]. Pilavaki et al. documented cough and tracheal deviation, whereas Melinte recorded dysphagia accompanied by thoracic pain [4,6]. Kola et al. noted the presence of growing chest discomfort and fatigue, whereas Muzurović’s patient had a cough and unusual chest pain [3,7]. Certain cases were asymptomatic and identified accidentally, exemplified by Regal et al.’s patient who exhibited mild dyspnea discovered during routine imaging [8]. El Haj et al. described a case of persisting respiratory symptoms for 4 months [9]. Other patients exhibited aberrant presentations, such as Mace et al.'s incidental magnetic resonance imaging (MRI) discovery and Imai et al.'s chronic cough erroneously assigned to another illness [2,10]. Wu et al. observed a patient with a productive cough without compressive symptoms [11]. Truong et al. and Walz et al. reported voice hoarseness, a pressure sensation exacerbated by palpation, and mild dysphagia [12,13]. These papers highlight the diagnostic difficulties associated with ETT.

Surgical excision is the preferred management, with modifications in the approach based on the mass's location, size, and proximity to vital structures. Our patient underwent a low cervical incision without sternotomy or manubriotomy. Other cases employed midline cervical incisions [2,12,13], transcervical resections, or robotic-assisted surgery [6,10]. Pilavaki’s and Wu’s patients underwent thoracotomy [4,11]. Sternotomy was needed for Nagireddy because of the tumor's closeness to important arteries, whereas Regal et al. executed a partial sternotomy [5,8]. Muzurović performed a combination sternotomy, thyroidectomy, parathyroidectomy, and thymectomy [7]. El Haj conducted uniportal video-assisted thoracoscopic surgery [9].

All cases reported full symptom resolution without complications postsurgery. Most subjects had normal postoperative imaging and TFTs. One patient needed L-thyroxine and oral calcium carbonate with close calcium, phosphate, and parathyroid hormone (PTH) monitoring [7]. Another case required minimal chest tube drainage [13].

Our effective application of a low cervical incision over more invasive options demonstrates the necessity of tailored surgical planning. However, the case's limitation is its single-patient nature, which limits generalizability and long-term follow-up. Future research should concentrate on identifying predictive factors for symptom development and optimizing minimally invasive approaches.

## Conclusion

This case underscores the significance of early detection and including ETT in the differentials of retrosternal or mediastinal tumors, especially when compressive symptoms are present. The presentation and management of ETT are typically consistent across age groups and genders; however, the young age of our patient introduces a distinctive aspect to this report. CT and MRI are critical for diagnosis, while biopsy confirms the benign nature of the mass. Surgical excision is the definitive treatment, providing symptom resolution and preventing complications. Familiarity with similar cases improves clinicians' comprehension of this uncommon condition, facilitating precise diagnosis and effective management approaches.

## Patient consent

Informed verbal consent was taken from the patient prior to publication.
